# Case of Hydrocephlus Successfully Treated by Iodide of Potassium

**Published:** 1847-03

**Authors:** 


*Case of Hydrocephlus successfully treated by Iodide of Potassium.* By LYMAN BRACKETT, M. D., of Rochester, Fulton co., Ind.

Josephine S. ætat 6 years, was seized on the first of April, 1846, with the usual symptoms of Hydrocephalus, which continued to progress, in defiance of the most active treatment, given with a view of checking the inflammation and preventing the effusion of serum, the symptoms of which have given the name of hydrocephalus to this truly obstinate and at times, fatal disease. The inflammation continued, and effusion took place (as indicated by the symptoms)

after the usual course had been steadily and perseveringly tried for the space of two weeks. During the last six days of this time she had been lying insensible to sight and sound; pupils very widely dilated and insensible to the strongest light. Continually rolling her head from side to side. Hemiplegia of right side, and partial paralysis of the left. Incessantly moaning, except when she would throw her left hand to her head, and cry out as if in great distress. This happened about every half-hour. Vomiting would almost invariable happen when she was raised in bed into a sitting posture. Involuntary passage of foeces and urine. Then after having tried all other customary remedies, I resolved on using the iodide potassium, knowing she could not long survive in her then condition.

I began by giving an aqueous solution of the iodide (composed of iodide of potassium ℈i to ℥ water,) gtt. xv. every three hours increasing the dose gradually to 30 gtt. An evident amendment was the next day perceptible, when some soreness of the mouth and bleeding of the gums took place. From thence forward she improved rapidly, and on the fourth day from commencing the iodide, I had the satisfaction of pronouncing her out of danger. The loss of power over the muscles of locomotion and of speech was not, however, perfectly relieved by it, but was restored by the epidermic application of a solution of strychnine along the course of the spinal column. The solution of strychnine was of the following composition:—Strychnine, grs. viii.; acetic acid ʒ i.; alcohol ℥ i: If you think the preceding case is worthy of a place in your Journal you will please publish it. I have made it as brief as possible that it might not occupy too much space.—*Illinois and Indiana Med. and Surg. Journal.*

